# Rationale for a Combination Therapy Consisting of MCL1- and MEK-Inhibitors in Acute Myeloid Leukemia

**DOI:** 10.3390/cancers11111779

**Published:** 2019-11-12

**Authors:** Katja Seipel, Karin Schmitter, Ulrike Bacher, Thomas Pabst

**Affiliations:** 1 Department for Biomedical Research (DBMR), University of Bern, Bern 3008, Switzerland; 2 Department of Medical Oncology, Inselspital, University Hospital Bern, Bern 3010, Switzerland; 3 Department of Hematology, Inselspital, University Hospital Bern, Bern 3010, Switzerland; veraulrike.bacher@insel.ch; 4 Center of Laboratory Medicine (ZLM), Inselspital, University Hospital Bern, Bern 3010, Switzerland

**Keywords:** acute myeloid leukemia (AML), FMS like tyrosine kinase 3 (FLT3), hematological malignancies, mitogen-activated protein kinase kinase (MEK; MAP2K; MAPKK), myeloid leukemia cell differentiation protein (MCL1), ribosomal protein S6 (RPS6), tumor suppressor p53 (TP53)

## Abstract

Amplification and overexpression of the myeloid cell leukemia differentiation protein MCL1 and the murine double minute protein MDM2 have been reported in various human tumors as well as hematological malignancies including acute myeloid leukemia (AML). While MCL1 is an anti-apoptotic member of the BCL-2 family proteins, MDM2 is an important cellular inhibitor of the p53 tumor suppressor. The key oncogene in AML is the FLT3 growth factor receptor gene. FLT3 signaling pathways including the MAPK cascade (RAS-RAF-MEK-ERK) are highly active in AML cells, leading to induced protein translation and cell proliferation as well as reduced apoptosis. Consequently, combined administration of MCL1-, MDM2-, and MEK-inhibitors may present a promising anti-leukemic treatment strategy. Here, we assessed the MCL1-antagonist S63845, the MDM2-inhibitor HDM201, and the MEK1/2-inhibitor trametinib as single agents and in combination in a variety of AML cell lines and mononuclear cells isolated from patients with hematological malignancies centered on myeloid leukemia, some lymphatic leukemia, as well as some lymphomas, for their ability to induce apoptosis and cell death. We observed a considerably varying anti-leukemic efficacy of the MCL1-inhibitor S63845 and the MEK1/2-inhibitor trametinib. Hematological cells with susceptibility to the single compounds as well as to the combined treatment were defined by elevated MCL1- and MEK-protein levels, independent of the mutational status of *FLT3* and *TP53*. Our data indicate that hematological cells with elevated MCL1- and MEK-protein levels are most sensitive to the combined treatment with S63845 and trametinib. MCL1- and MEK1/2-protein expression may be valid biomarkers for treatment response to S63845 and trametinib, respectively.

## 1. Introduction

Tumor cells have evolved mechanisms to prevent induction of apoptosis. In hematological malignancies including acute myeloid leukemia (AML) tumor cells prevent apoptosis by elevated expression of anti-apoptotic proteins of the BCL2 family. The induced myeloid leukemia cell differentiation protein 1 (MCL1), an anti-apoptotic member of the BCL2 family proteins, is often upregulated in AML cells, particularly at relapse [1]. MCL1 exerts its anti-apoptotic function by sequestering pro-apoptotic proteins BAK and BAX [2]. 

S63845 is a new selective MCL1-inhibitor with potential use across a number of hematological malignancies including myeloma, AML, and lymphoma [3]. Kotschy et al. performed combinational studies with a variety of kinase inhibitors and found that in all cases these kinase inhibitors enhanced S63845 efficacy. Moreover, combining BH3-mimetics to target both BCL-2 and MCL1 had potent activity in pre-clinical models of acute myeloid leukemia [4] and in T-cell acute lymphoblastic leukemia cells [5]. 

The key oncogene in AML is the FMS-kinase 3 (*FLT3*) gene. This growth factor receptor is often overexpressed in AML cells with a normal *FLT3* gene [6] or constitutively active in AML cells with *FLT3* mutation [7]. FLT3 receptor signaling promotes cell survival and prevents apoptosis via activation of the PI3K-PDK1-AKT kinase cascade and the MAP kinase cascade (MEK-ERK-mTOR) [8]. A number of chemical compounds with varying specificity against MEK kinases have been developed and are currently evaluated in preclinical and clinical cancer trials [9]. Trametinib (mekinist) and cobimetinib are MEK-specific inhibitors effective against metastatic melanoma carrying the BRAF V600E mutation [10], which may also be effective in the treatment of acute myeloid leukemia [11]. 

The key tumor suppressor in AML is the *TP53* gene. The p53 protein levels are very low in AML cells due to overexpression of the cellular p53 inhibitor MDM2 [12]. Pharmacological activation of wildtype p53 levels is a promising approach in the therapy of leukemia [13]. A number of chemical MDM2-inhibitors have been developed and are currently evaluated in clinical trials [14,15].

Here we investigated the MCL1 inhibitor S63845, the MEK-inhibitor trametinib, and the MDM2- inhibitor HDM201 in AML cell lines and patient cells in order to identify a potentially effective treatment for AML patients unfit for intensive chemotherapy. The study might provide the rationale for initiating a clinical study evaluating this treatment combination.

## 2. Materials and Methods

### 2.1. Cell Lines

OCI-AML2 (*FLT3*wt, *TP53*wt), OCI-AML3 (*FLT3*wt, *NPM1* L287fs, *NRAS* Q61L, *TP53*wt), MOLM-13 (*FLT3*ITD, t(9;11), *TP53*wt), and MOLM-16 (*FLT3*wt, *TP53* V173M, *TP53* C238S) cell lines were supplied by the Leibniz Institute DSMZ, German Collection of Microorganisms and Cell Cultures. AML cells were grown in RPMI 1640 (SIGMA-ALDRICH, St. Louis, MO, USA) supplemented with 20% fetal bovine serum (FBS, Biochrom GmbH, Germany). We passaged the cells for a maximum of twelve times. 

### 2.2. Patient Samples

Mononuclear cells of 22 patients with hematological malignancies diagnosed and treated at the University Hospital, Bern, Switzerland between 2015 and 2018 were included in this study. Informed consent from all patients was obtained according to the Declaration of Helsinki, and the studies were approved by the local ethics committee of Bern, Switzerland, decision number #221/15. Mutational screening for *FLT3, NPM1*, and *TP53* genes were performed for all AML samples. Conventional karyotype analysis of at least 20 metaphases were performed for all hematological samples. Peripheral blood mononuclear cells (PBMCs) and bone marrow mononuclear cells (BMMCs) were collected at the time of diagnosis before initiation of treatment. 

### 2.3. Cytotoxicity Assays

AML cells were treated with compound diluent only (controls) or with the MCL1-inhibitor S63845 (HY-100741, MedChemExpress, Monmouth Junction, NJ, USA), the MDM2-inhibitor NVP-HDM201 (Novartis, Switzerland), and the MEK1/2-inhibitor trametinib (HY-10999A, MedChem Express, Monmouth Junction, NJ, USA). Cell viability was determined using the MTT-based in vitro toxicology assay (SIGMA-ALDRICH, St. Louis, MO, USA). For AML cell lines, four independent assays (biological replicates) with four measurements (technical replicates) per dosage were performed. For hematological patient samples two independent assays with three technical replicates per dosage were performed. Data were analyzed on GraphPad Prism software using Mann–Whitney tests and are depicted as average values with standard deviation on column graphs.

### 2.4. Calculation of Combination Index

Combination indexes were calculated on CompuSyn software (version 1.0; ComboSyn, Inc. Paramus, NJ, USA), according to the method of Chou and Talalay [16,17]. For combination index values of cytotoxicity effects, the average fraction of life cells (0.11–0.99) in the cytotoxicity assays were used. 

### 2.5. Imaging Cytometry

Imaging cytometry was done on the NC-3000 cell analyzer (ChemoMetec, Allerod, Denmark) with reagents supplied by ChemoMetec. To determine induction of cell death, apoptotic cells were stained with AnnexinV-CF488A conjugate (Biotium, Fremont, CA, USA) in AnnexinV buffer and Hoechst 33342 (10 μg /mL) for 15 min at 37 °C, followed by several washes. Three independent assays with two technical replicates per dosage were performed. Data were analyzed on Graphpad Prism software using Mann–Whitney tests and are depicted as column graphs with average values and standard deviation. 

### 2.6. Measurement of Protein Levels by Western Blot

Total protein extracts were prepared by cell lysis in radioimmunoprecipitation assay buffer (RIPA lysis). A total of 100 μg total protein extracts were separated on polyacrylamid gel by electrophoresis (PAGE), transferred to a nitrocellulose membrane and incubated with mouse anti-phospho-S6 (Ser235/236) cupk43k (#14-9007-82, Invitrogen, Thermo Fisher Scientific), mouse anti-actin IgG (MAB 1501, Millipore, CA), followed by IRDye^®^ 680LT goat anti-mouse IgG (LI-COR Biotechnology, BadHomburg, Germany). Membranes were scanned on a LI-COR Odyssey Infrared Scanner (LI-COR Biotechnology, Germany). Bands were quantified on Image Studio software. Three independent assays were performed. One representative blot is depicted. 

### 2.7. Enzyme-Linked Immunosorbent Assay (ELISA)

Protein extraction was done according to standard protocol. In short, cell pellets were lysed in RIPA buffer. GAPDH, FLT3, MCL1, MEK1, and MEK2 protein levels were determined with double-antibody Sandwich ELISA (SEB932Hu, SEA039Hu, SEC615Hu, SED559H1, Cloud-Clone Corp., Houston, TX, USA; E10344h, EIAab science Inc, Wuhan, China). FLT3, MCL1, and MEK1/2 values were normalized with GAPDH values. Two independent assays with three technical replicates were performed per sample. Statistical analysis was performed using averaged normalized values and the Mann–Whitney test or linear regression on GraphPad Prism software. Data are depicted as scatter plots with median values, or as XY graphs with linear regression.

## 3. Results

### 3.1. Synergistic Effects on Cell Viability in AML Cell Lines Treated with the MCL1 Inhibitor S63845 and the MEK Inhibitor Trametinib or the MDM2 Inhibitor HDM201

To determine the sensitivity of AML cells to S63845, HDM201, and trametinib, AML cell lines were studied in in vitro cytotoxicity assays. The cell lines covered the major molecular AML subtypes characterized by *FLT3*-ITD and *FLT3* wildtype, *NPM1* mutant and wildtype, as well as *TP53* mutant and wildtype genes. 

**Figure 1:**
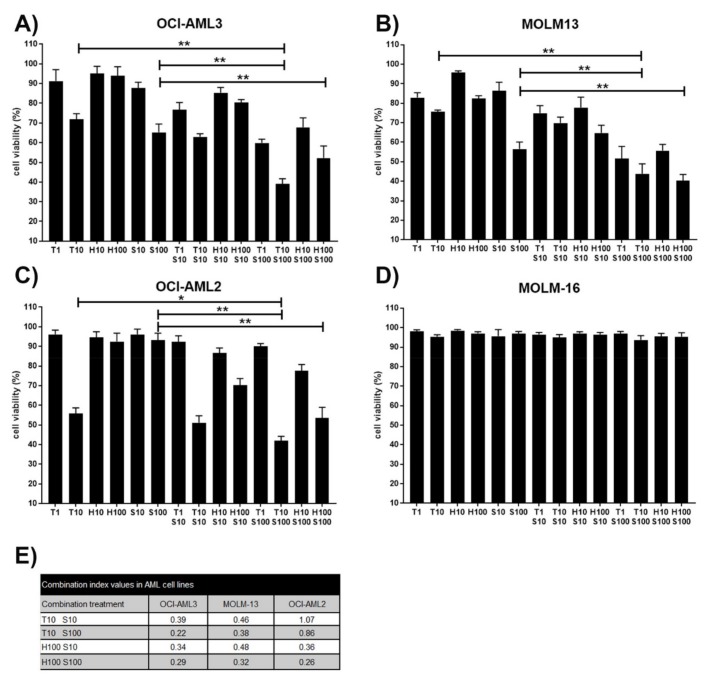
Susceptibility of AML cells to S63845, HDM201, and trametinib. Cell viability in AML cell lines OCI-AML3 (**A**), MOLM-13 (**B**), OCI-AML2 (**C**), and MOLM-16 (**D**) treated for 20 h with increasing dosages 1nM, 10nM, and 100nM of S63845 (S), HDM201 (H), and trametinib (T) alone or in combination. All values are in reference to mock treated cells (= 100% viability). Combination index values (**E**) were calculated on ComboSyn software. Combinatorial effects were interpreted to correspond to strong synergism for CI= 0.1–0.3; distinct synergism for CI = 0.3–0.7, mild synergism for CI = 0.7–0.9; additive for CI = 0.9–1.1. Significance is denoted for *p* < 0.05 (*); *p* < 0.005 (**).

AML cells lines varied considerably with respect to S63845, HDM201, and trametinib sensitivity (Figure 1). MOLM-13 and OCI-AML3 cells were susceptible to 100nM S63845 with 60–70% viable cells after 20 h, while MOLM-16 and OCI-AML2 cells were not susceptible to 100nM S63845 with 95% cell viability. OCI-AML2 were most susceptible to trametinib, with 50% viable cells after 20 h, MOLM-13 and OCI-AML3 cells were susceptible with 70–80% viable cells, and MOLM-16 cells had no reduction in cell viability. OCI-AML2 and MOLM-13 cells were susceptible to 100nM HDM201 with 80% viable cells after 20 h, while OCI-AML3 and MOLM-16 showed no reduction in cell viability. 

Synergistic effects on cell viability were detected in the combination treatments with S63845 and trametinib in OCI-AML3 and MOLM-13 cells and in the combination treatments with S63845 and HDM201 in OCI-AML2, OCI-AML3, and MOLM-13 cells with combination indexes of 0.2 to 0.5. Synergism was calculated to be strong in OCI-AML3 cells for the combination of 100nM S63845 and 10nM trametinib or 100nM HDM201 with combination index values of 0.2 to 0.3. In direct comparison trametinib had hundredfold greater potency compared to HDM201, as 1nM trametinib and 100nM HDM201 had the same effect on MOLM-13 cells. Hence, the later studies in malignant cells of hematological patients were focused on the combination treatment with trametinib and S63845.

### 3.2. Induction of Apoptosis in AML Cell Lines Treated with the MCL1 Inhibitor S63845 and the MEK Inhibitor Trametinib or the MDM2 Inhibitor HDM201

**Figure 2:**
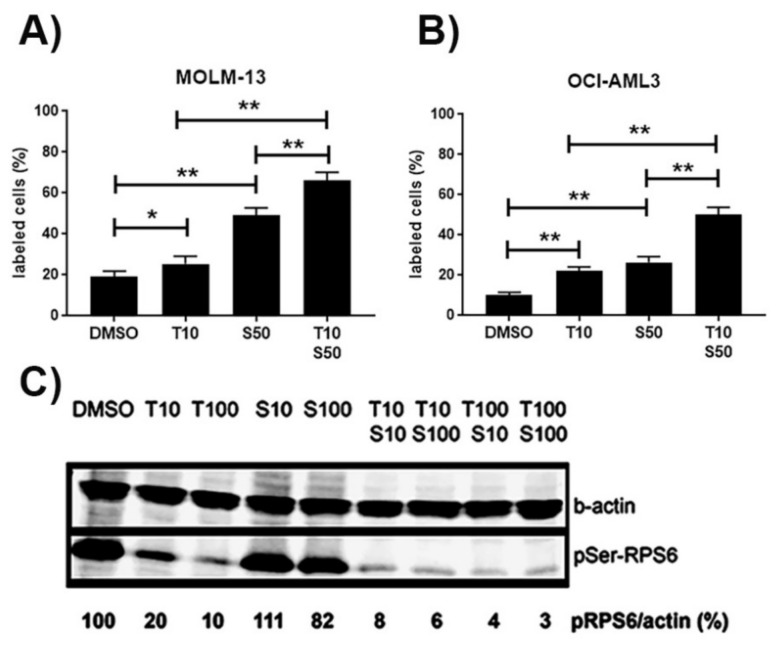
Cellular and molecular effects in AML cells treated with S63845, HDM201, and trametinib. Increase in the percentage of annexin positive apoptotic cells based on cytometric analysis in the AML cell lines MOLM-13 (**A**) and OCI-AML3 (**B**) treated for 20 h with single compounds or with combination treatment. Reduced levels of phospho-Ser-RPS6 (**C**) in OCI-AML3 cells treated with the indicated amounts of S63845 (S, nM) and trametinib (T, nM). Representative Western blot of total protein extracts detecting pSer-RPS6 and β-actin. Significance is denoted for *p* < 0.05 (*); *p* < 0.005 (**).

To determine whether the loss of cell viability was due to induction of apoptosis, AML cell lines were studied in cytometric assays. The number of apoptotic cells was determined by annexin V staining. Apoptosis was induced in MOLM-13 and OCI-AML3 cells treated with S63845 and trametinib (Figure 2A,B). S63845 concentration had to be reduced to 50 nM as MOLM-13 cells induced massive apoptosis at 100 nM. Numbers of apoptotic cells were maximal after combined treatment, indicating an enhanced induction of apoptosis in cells treated with S63845 and trametinib. MEK inhibition may also lead to reduced phosphorylation of ribosomal protein S6 (RPS6) [18]. In addition to apoptosis induction, we detected reduced phosphorylation of RPS6 protein in OCI-AML3 cells treated with the MEK-inhibitor trametinib (Figure 2C). Minimal amounts of pSer-RPS6 were present after combined treatment, indicating an enhanced reduction of pSer-RPS6 in cells treated with S63845 and trametinib. 

**Figure 3:**
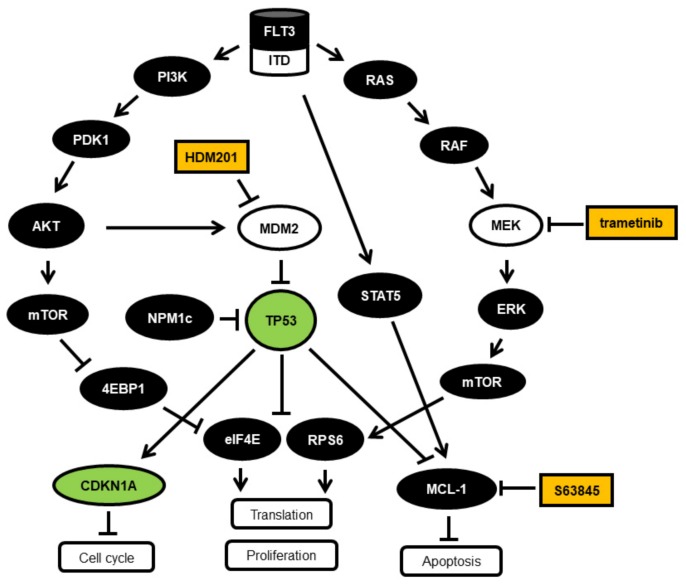
Schematic representation of the FLT3 signaling pathways and downstream effects. The FLT3 growth factor receptor signaling is mediated by PI3K-PDK1-AKT and RAS-RAF-MEK-ERK, leading to cell growth and proliferation involving p53 inhibition and *MCL1* gene induction. p53 function can be reactivated by HDM201 treatment, leading to inhibition of *MCL1* gene expression. MCL1 function can be blocked by S63845. FLT3-ITD is a constitutively active growth factor receptor signaling via PI3K-AKT [8], via RAS-MEK-ERK [19], and via STAT5 [20], leading to cell growth and proliferation via p53 inhibition and MCL1 induction.

In summary, these data suggest that treating AML cells with the MCL1-inhibitor S63845 and the MEK-inhibitor trametinib leads to reduced levels of phospho-Ser-RPS6 protein, to inhibition of translation, and induction of apoptosis, as summarized in Figure 3. 

### 3.3. Varying Sensitivity of Hematological Patient Cells to Treatment with the MCL1 Inhibitor S63845 and the MEK Inhibitor Trametinib

**Figure 4:**
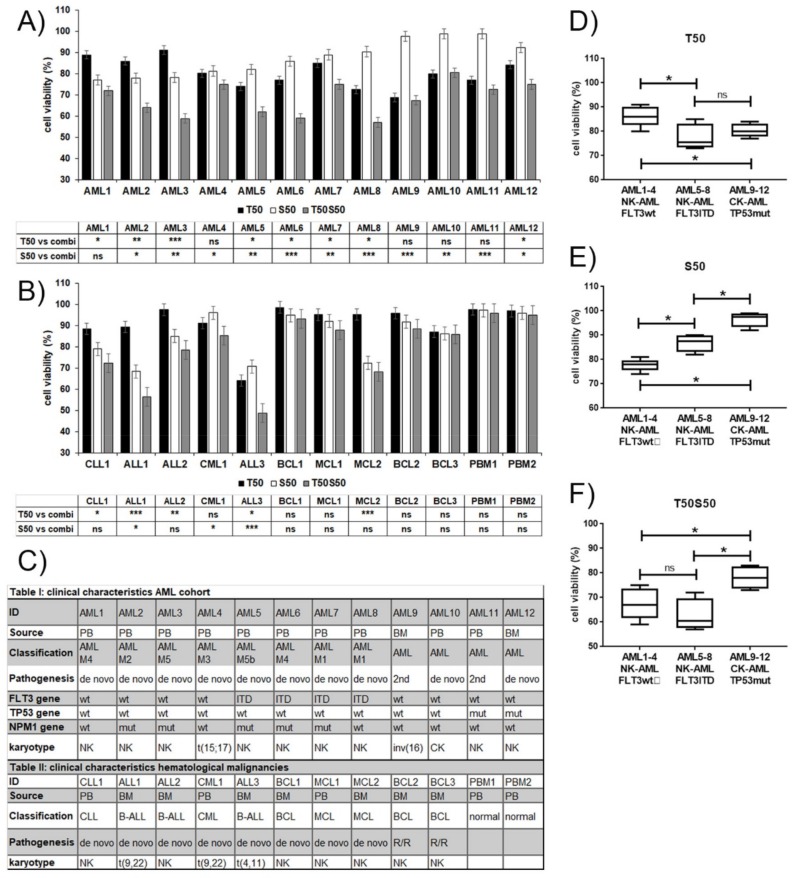
Hematological cells response to S63845, trametinib, and combination treatment. Cell viability assessments in mononuclear cells of AML (**A**), other hematological malignancies (**B**), and normal cells treated for 20 h with S63845 and trametinib alone or in combination. All values are in reference to mock treated cells (= 100% viability). Statistical significance is indicated for the effects of single versus combination treatments. (**C**) Clinical characteristics of the AML and other hematological malignancies. Acute myeloid leukemia (AML), acute lymphatic leukemia (ALL), chronic lymphatic leukemia (CLL), chronic myeloid leukemia (CML), B-cell lymphoma (BCL), mantle cell lymphoma (MCL). Peripheral blood (PB), bone marrow (BM), normal karyotype (NK), complex karyotype (CK). Relapsed/refractory (R/R). Susceptibility of grouped AML subsets to trametinib (**D**), S638 (**E**), and combination treatment (**F**). Significance is denoted for *p* < 0.05 (*); *p* < 0.005 (**); *p* < 0.0005 (***); *p* > 0.05 (ns).

Considering the wide range of response observed in AML cell lines to the combination treatment with S63845 and trametinib, we expected a varied susceptibility to this treatment among AML patient cells, and hypothesized that susceptibility may extend to a wider range of hematological malignant cells. We performed in vitro cytotoxicity assays using mononuclear cells isolated from peripheral blood or bone marrow of patients with hematological malignancies centered on twelve acute myeloid leukemias (AML1-12) (Figure 4A). Cytotoxicity assays were also performed on other leukemic cells, including one chronic myeloid leukemia (CML), three acute and one chronic lymphocytic leukemias (ALL, CLL), and five lymphomas including three B-cell lymphomas (BCL) and two mantle cell lymphomas (MCL) (Figure 4B). The AML blast cells covered the major molecular subtypes, including *FLT3* wildtype and *FLT3*-ITD*, TP53* wildtype and mutated, de novo, and secondary AML (Figure 4C). The threshold of susceptibility was deliberately set at median levels of cell viability. The responses to S63845 and trametinib varied among the tested hematological samples. Half of the AML samples (AML 2, 3, 5, 6, 8, 9) were susceptible to the combination treatment with less than 70% viability after 20 h. Compared to *FLT3* wildtype NK-AML (AML 1–4), the *FLT3*-ITD positive NK-AML (AML 5–8) appeared to be more susceptible to trametinib (Figure 4D), and less susceptible to S63845 (Figure 4E). The susceptibility to the combination treatment was greater in the NK-AML cells than in the CK-AML or TP53 mutated AML (Figure 4F). The AML cells least susceptible to S63845 included one CK-AML and two secondary AML (AML 9, 10, 11), which, however, were susceptible to trametinib. Of the other hematological samples, two ALL samples (ALL1, ALL3) were susceptible to the combination treatment. One MCL sample (MCL2) was susceptible to S63845. None of the BCL samples were susceptible to the combination treatment. Normal peripheral blood monocytes were only minimally affected.

### 3.4. MCL1 and MEK Protein Levels as Biomarkers for Treatment Response to S63845 and Trametinib

**Figure 5:**
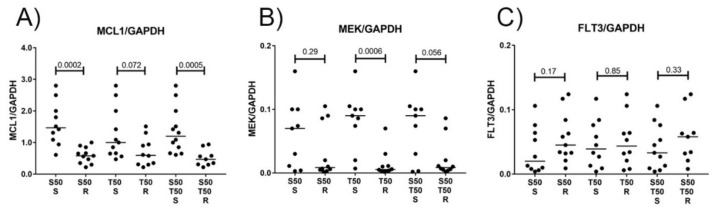
MCL1 and MEK1 protein levels are biomarkers for S63845 and trametinib treatment response in mononuclear cells isolated from patients with hematological malignancies. Relative protein levels of MCL1/GAPDH(**A**), MEK/GAPDH (**B**), and FLT3/GAPDH (**C**) in mononuclear cells of 16 patients with hematological malignancies and 4 AML cell lines in correlation to susceptibility of hematological cells treated with 50nM S63845 (S50), 50nM trametinib (T50), or combination treatment (S50T50) for 20 h. Hematological cells with elevated MCL1 protein levels are susceptible to S63845, while hematological cells with elevated MEK protein levels are susceptible to trametinib. There is no correlation of FLT3 protein levels to susceptibility of hematological cells to S63845 or trametinib. The threshold of sensitivity was deliberately set at median levels of cell viability. Cells responsive at levels above/below the threshold were called susceptible (S) and resistant (R).

**Figure 6:**
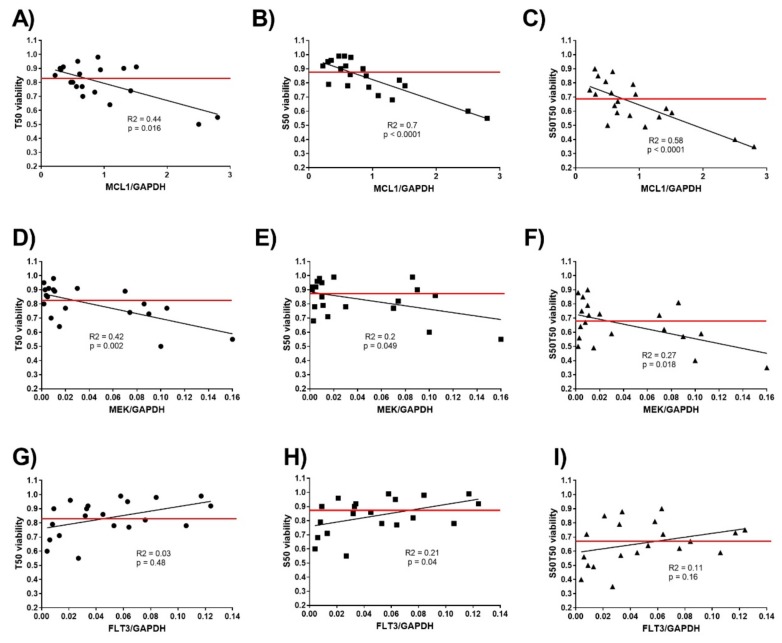
Correlation of viability and biomarker expression in mononuclear cells isolated from 16 patients with hematological malignancies and 4 AML cell lines treated with S63845 and trametinib. Relative protein levels of MCL1/GAPDH (**A**–**C**), MEK/GAPDH (**D**–**F**), and FLT3/GAPDH (**G**–**I**) in mononuclear cells of patients with hematological malignancies in correlation to cell viability after 20-hour treatment with 50nM S63845 (S50), 50nM trametinib (T50), or combination treatment (S50T50). Hematological cells with elevated MCL1-protein levels are susceptible to S63845 and trametinib, while hematological cells with elevated MEK-protein levels are susceptible to trametinib. The threshold of sensitivity (red line) was deliberately set at median levels of cell viability.

Susceptibility to targeted therapies should, by definition, correlate to the abundance of the target proteins in the hematological cells. We therefore determined the MCL1, MEK1, and MEK2 protein levels in hematological cells and AML cell lines before treatment. MCL1 protein levels ranged from 0.2 to 2.8 MCL1/ GAPDH, with elevated MCL1 levels in cells more susceptible to 50nM S63845 with median values of 1.4 versus 0.6 MCL1/GAPDH (*p* = 0.0005) (Figure 5A; Figure 6A–C). AML cell lines MOLM-13 and OCI-AML3, which were susceptible to 50nM S63845, had elevated MCL1 levels of 2.8 and 2.5 MCL1/GAPDH. AML cell lines OCI-AML2 and MOLM-16, which were resistant to S63845, had reduced MCL1 levels of 0.5 and 0.3 MCL1/GAPDH. MCL1 protein levels were also elevated in cells susceptible to the combination treatment with median values of 1.2 versus 0.5 (*p* = 0.0004). MEK1 protein levels ranged from 0.002 to 0.16 MEK1/GAPDH, while MEK2 protein levels ranged from 0.001 to 0.02 MEK2/GAPDH. Elevated MEK levels were present in cells susceptible to 50nM trametinib, with median values of 0.09 versus 0.006 MEK/GAPDH (*p* = 0.0006) (Figure 5B; Figure 6D–F). AML cell lines MOLM-13 and OCI-AML3, which were susceptible to trametinib, had elevated MEK levels of 0.1 and 0.16 MEK/GAPDH, while OCI-AML2 and MOLM-16, both unsusceptible to trametinib, had low MEK levels of less than 0.01 MEK/GAPDH. As FLT3 signaling activity may correlate to MEK activity, we also determined the FLT3 protein levels in all hematological samples in the present study. FLT3 protein levels ranged from 0.004 to 0.14 FLT3/GAPDH (Figure 5C; Figure 6G–I). There was, however, no correlation of FLT3 protein levels with susceptibility to S63845 or trametinib. This is in contrast to results of a study on AML treatment with another MEK inhibitor, cobimetinib, where the abundance of FLT3 protein in AML cells correlated to cobimetinib susceptibility [21]. The biomarkers for the response to the treatment with the MEK- inhibitor trametinib may therefore be different from the biomarkers for response to cobimetinib. 

## 4. Discussion

The aim of this study was to identify subsets of patients with hematological malignancies susceptible to the combined treatment with the MCL1 antagonist S63845 in combination with the MDM2-inhibitor HDM201 or the MEK-inhibitor trametinib. The three inhibitors were assessed as single agents and in combination in a variety of AML cell lines. In direct comparison trametinib had a hundredfold greater potency compared to HDM201, as 1nM trametinib and 100nM HDM201 had the same effect on MOLM-13 cells. Hence, the studies in malignant cells of hematological patients were focused on the combination treatment with trametinib and S63845. Hematological cells characterized by elevated MCL1-protein levels were susceptible to S63845 treatment, while cells with elevated MEK protein levels were susceptible to trametinib. Consequently, susceptibility to the combination treatment with S63845 and trametinib was greater in hematological cells with elevated MCL1 and MEK levels. Thus MCL1 and MEK protein expression levels in the leukemic cells may be response predictors in combination treatments with S63845 and trametinib. The significance of the in vitro susceptibilities would have to be corroborated in a clinical phase I study, as effective drug concentrations may be different in vivo.

The molecular mechanisms underlying the correlation of MCL1- and MEK1-protein levels and response to S63845 and trametinib may be related to the phenomenon of oncogene addiction, where the tumor relies on a dominant oncogene or mechanism for growth and survival, so that inhibition of this specific oncogene/pathway is sufficient to halt the neoplastic phenotype [22,23]. The MEK- inhibitors cobimetinib and trametinib appear to target different subsets of hematological cells. While susceptibility to cobimetinib appears to correlate to FLT3-protein levels [21], susceptibility to trametinib does not correlate to FLT3, but to MEK1/2-protein levels. The reason for this cell-type specificity may be related to the target specificity of the two compounds. Cobimetinib and trametinib are highly selective, allosteric, and non-ATP competitive MEK1/2-inhibitors. Cobimetinib binds to phosphorylated MEK1, with a hundredfold greater potency for MEK1 over MEK2, while trametinib binds to non-phosphorylated MEK1 and MEK2 with similar potencies, though both inhibit downstream ERK signaling [10]. In contrast to trametinib, cobimetinib may also target AKT and PKC kinases [24]. As FLT3 signals via both MEK1/2 and AKT, this may explain the correlation of FLT3-protein levels to cobimetinib susceptibility. 

Different combinations of targeted therapies emerge to be suitable for the treatment of specific subsets of AML patients. While NK-AML cells with mutated *FLT3* appear to be susceptible to FLT3- and MDM2-inhibitors, in particular the FLT3 inhibitor midostaurin and the MDM2-inhibitor HDM201 [25], NK-AML cells with elevated FLT3 protein levels appear to be most susceptible to the MEK inhibitor cobimetinib and the MDM2-inhibitor idasanutlin [21]. Here we found that the MCL1-inhibitor S63845 and the MEK-inhibitor trametinib have target specificity for primary cells of hematological malignancies with elevated MCL1- and MEK-protein levels. For cells with low MEK-protein levels, other combination therapies may be more effective, as e.g., the combination of the MCL1-inhibitor S63849 and the MDM2-inhibitor HDM201, which had highly synergistic effects on OCI-AML2 cells in this study. It may also be of value to have several treatment options which may be applied in series. For patients with highly aggressive *FLT3*-ITD positive AML, a combination of FLT3- and MDM2-inhibitor may be a justified first line therapy, which could be followed by a second line therapy with a combination of MCL1- and MEK-inhibitor. 

## 5. Conclusions

The MCL1 inhibitor S63845 and the MEK inhibitor trametinib may be effective therapeutic compounds in acute myeloid leukemia. Here we found that the MCL1-inhibitor S63845 and the MEK-inhibitor trametinib specifically target primary cells of acute myeloid leukemia with elevated MCL1- and MEK-protein levels. MCL1- and MEK1/2-protein expression may be valid biomarkers for treatment response to S63845 and trametinib, respectively. 

## Abbreviations

acute myeloid leukemia (AML); acute lymphoblastic leukemia (ALL); chronic lymphoblastic leukemia (CLL); chronic myeloid leukemia (CML); FMS like tyrosine kinase 3 (FLT3); glyceraldehyde 3-phosphate dehydrogenase (GAPDH); internal tandem duplication (ITD); mouse double minute 2 homolog (MDM2); mitogen-activated protein kinase kinase (MEK); normal karyotype (NK); nucleophosmin (NPM1); tumor suppressor p53 (TP53); wildtype (wt); mutated (mut)
